# Analysis of the three-dimensional anatomical variance of the distal radius using 3D shape models

**DOI:** 10.1186/s12880-017-0193-9

**Published:** 2017-03-09

**Authors:** Sebastian F. Baumbach, Jakob Binder, Alexander Synek, Fabian G. Mück, Yan Chevalier, Ekkehard Euler, Georg Langs, Lukas Fischer

**Affiliations:** 1 Department of General, Trauma and Reconstructive Surgery, University Hospital LMU Munich, Nussbaumstr. 20, Munich, 80336 Germany; 2 Institute of Lightweight Design and Structural Biomechanics, Vienna University of Technology, Getreidemarkt 9, Vienna, 1060 Austria; 3 Department of Clinical Radiology, University Hospital LMU Munich, Nussbaumstr. 20, Munich, 80336 Germany; 4 Department of Orthopaedic Surgery, Physical Medicine and Rehabilitation, University Hospital LMU Munich, Campus Grosshadern, Marchioninistraße 15, Munich, 81377 Germany; 5 Computational Imaging Research Laboratory, Department of Biomedical Imaging and Image-guided Therapy, Medical University of Vienna, Waehringer Guertel 18-20, Vienna, 1090 Austria; 6 Software Competence Center Hagenberg GmbH, Softwarepark 21, Hagenberg, 4232 Austria

**Keywords:** Image processing, Anatomical model, Distal radius, Statistical model, Computed tomography

## Abstract

**Background:**

Various medical fields rely on detailed anatomical knowledge of the distal radius. Current studies are limited to two-dimensional analysis and biased by varying measurement locations. The aims were to 1) generate 3D shape models of the distal radius and investigate variations in the 3D shape, 2) generate and assess morphometrics in standardized cut planes, and 3) test the model’s classification accuracy.

**Methods:**

The local radiographic database was screened for CT-scans of intact radii. 1) The data sets were segmented and 3D surface models generated. Statistical 3D shape models were computed (overall, gender and side separate) and the 3D shape variation assessed by evaluating the number of modes. 2) Anatomical landmarks were assigned and used to define three standardized cross-sectional cut planes perpendicular to the main axis. Cut planes were generated for the mean shape models and each individual radius. For each cut plane, the following morphometric parameters were calculated and compared: maximum width and depth, perimeter and area. 3) The overall shape model was utilized to evaluate the predictive value (leave one out cross validation) for gender and side identification within the study population.

**Results:**

Eighty-six radii (45 left, 44% female, 40 ± 18 years) were included. 1) Overall, side and gender specific statistical 3D models were successfully generated. The first mode explained 37% of the overall variance. Left radii had a higher shape variance (number of modes: 20 female / 23 male) compared to right radii (number of modes: 6 female / 6 male). 2) Standardized cut planes could be defined using anatomical landmarks. All morphometric parameters decreased from distal to proximal. Male radii were larger than female radii with no significant side difference. 3) The overall shape model had a combined median classification probability for side and gender of 80%.

**Conclusions:**

Statistical 3D shape models of the distal radius can be generated using clinical CT-data sets. These models can be used to assess overall bone variance, define and analyze standardized cut-planes, and identify the gender of an unknown sample. These data highlight the potential of shape models to assess the 3D anatomy and anatomical variance of human bones.

**Electronic supplementary material:**

The online version of this article (doi:10.1186/s12880-017-0193-9) contains supplementary material, which is available to authorized users.

## Background

Various medical fields rely on detailed anatomical knowledge of the distal radius. This is required to understand joint kinematics, improve fracture pattern analysis, plan surgical procedures [1, 2], design novel osteosynthetic devices [3–5], and identify human remains [6, 7]. Up to now, literature on the anatomy of the distal radius is limited to morphologic (shape) and morphometric (size) studies based on radiographs [8] or single computed tomography (CT) slices [9–11]. Limiting the analysis to two-dimensional (2-D) results has two predominant limitations: first, it does not allow the display of three-dimensional (3D) shape variation of the distal radius; second, the use of single CT slices may impair inter-subject comparability due to inconsistent slice position and orientation.

A well-established methodology in assessment of 3D anatomy and anatomical variances of bones are statistical shape models. These can be generated from a database of CT scans. Following 3D surface segmentation, a dense set of corresponding surface landmarks is generated for each bone. Based on this information, 3D shape models can be calculated and the variation of each surface point within the population illustrated. These variations are referred to as modes. A further application of these 3D shape models is the generation of two-dimensional (2D) slices of each bone within the database with identical matching location and orientation. Finally, the 3D shape models can be used to classify anatomical geometries into groups, for instance to determine gender of unidentified bones.

Statistical shape models have been applied for segmentation of vertebra [12], femora [13, 14], or brain structures [15]. Van Giessen and colleges [16] used this methodology to analyze wrist bone motion patterns. No study has yet applied this methodology to assess the general 3D anatomy and population based variation of the distal radius, or generated inter-specimen consistent (position and orientation) cut planes. Therefore, the primary aim of this study was to generate and analyze a statistical 3D shape model of the intact distal radius. Specifically, 3D shape models were generated to 1) investigate the 3D shape variation, 2) generate standardized cut planes and evaluate morphometric parameters, and 3) test the model’s classification accuracy for radius side and gender.

## Methods

### Study design

A retrospective, CT-based image processing study was designed to investigate anatomical variance of the distal radius. It was organized in three steps: First, 3D shape models were generationed; Second, corresponding, uniform cut-planes were computed and morphometric parameters analized; Third, the model’s classification accuracy for side and gender was assessed. The local ethics committee approved the study (Ref. Nr. 126-13).

### Patient identification

Consecutive CT-scans of intact radii were identified using the local radiographic database (University Hospital LMU Munich). The search period was 2 years, the search terms used were: CT-scan AND wrist OR scaphoid; Eligibility criteria were 18 year of age, sufficiently large region of interest, identical scan and reconstruction parameters (i.e. identical scan protocol, bone kernel reconstructions, 1.25 mm axial slice thickness; Discovery HD 750, GE Healthcare, Waukesha IL/USA), no signs of current/previous fractures or morphologic changes such as osteoarthritis, bone cysts or tumors. If both radii of one person were eligible, only the right radius was included. No sample size calculation could be conducted due to missing preliminary data. Previous studies investigating the volar cortical angle of the distal radius included 74 ± 23 patients on average [9–11, 17–19]. Therefore, we aimed at a study population of 90 radii (mean + 1SD) and a gender ratio of 50% female.

### 3D shape model generation and analysis

**Fig. 1:**
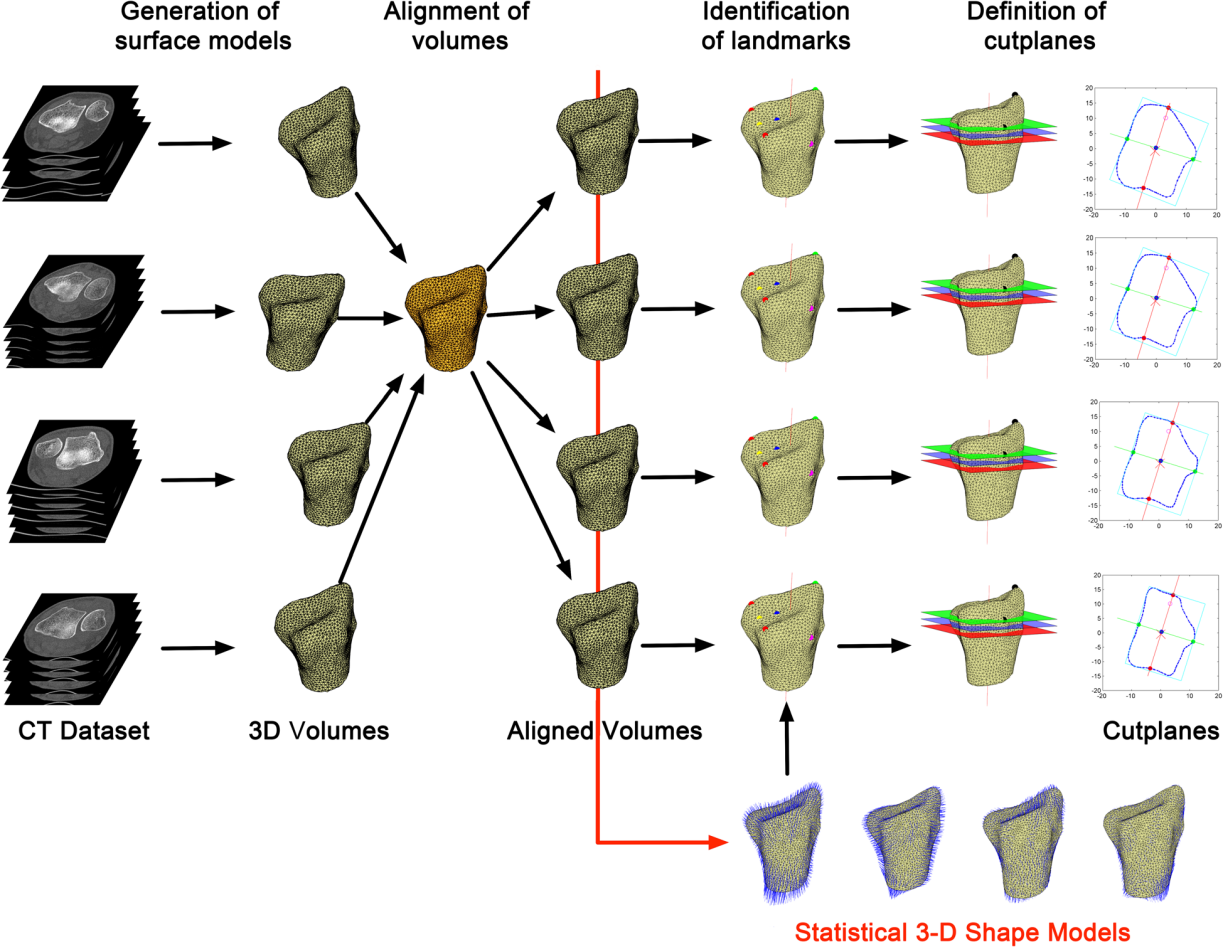
Overall workflow

The DICOM datasets of all distal radii were anonymized. The general workflow is outlined in Fig. 1. The CT-images (Fig. 1 3D Volumes) were segmented manually (Slicer 3D, [20]). Triangulated surfaces (0.1 mm side length, according to [21]) were generated using custom Python codes and CGAL libraries [22]. These were then registered to one radius to achieve a uniform alignment of the surface models (Fig. 1 Alignment). A Point Distribution Model (PDM) [23] was used to construct the shape model by computing the significant eigenmodes and thereby the shape variation of the data set. These shapes consisted of a set of *n* 3-dimensional landmarks. Correspondences between the shape landmarks were derived utilizing minimum description length (MDL) [24]. The shape information was then used to build models (Fig. 1 Statistical 3-D Shape Models), which allowed representation of the original shapes and generalization of new shapes within the distribution of shapes in the data set. The detailed process is outlined in Additional file 1. Three shape models were generated for all radii, as well as separately for gender and side. The gender and side specific models were used to analyze the general morphology of the distal radius. The 3D shape variation was assessed by evaluating the number of modes of the side and gender specific shape models. The number of modes was determined based on a 95% explained variance threshold. The first 5 modes were plotted. All calculations were performed in Matlab R2015a (The MathWorks, Inc., Natick, Massachusetts, United States).

### Standardized cross-sectional cut planes and morphometric parameters

**Fig. 2:**
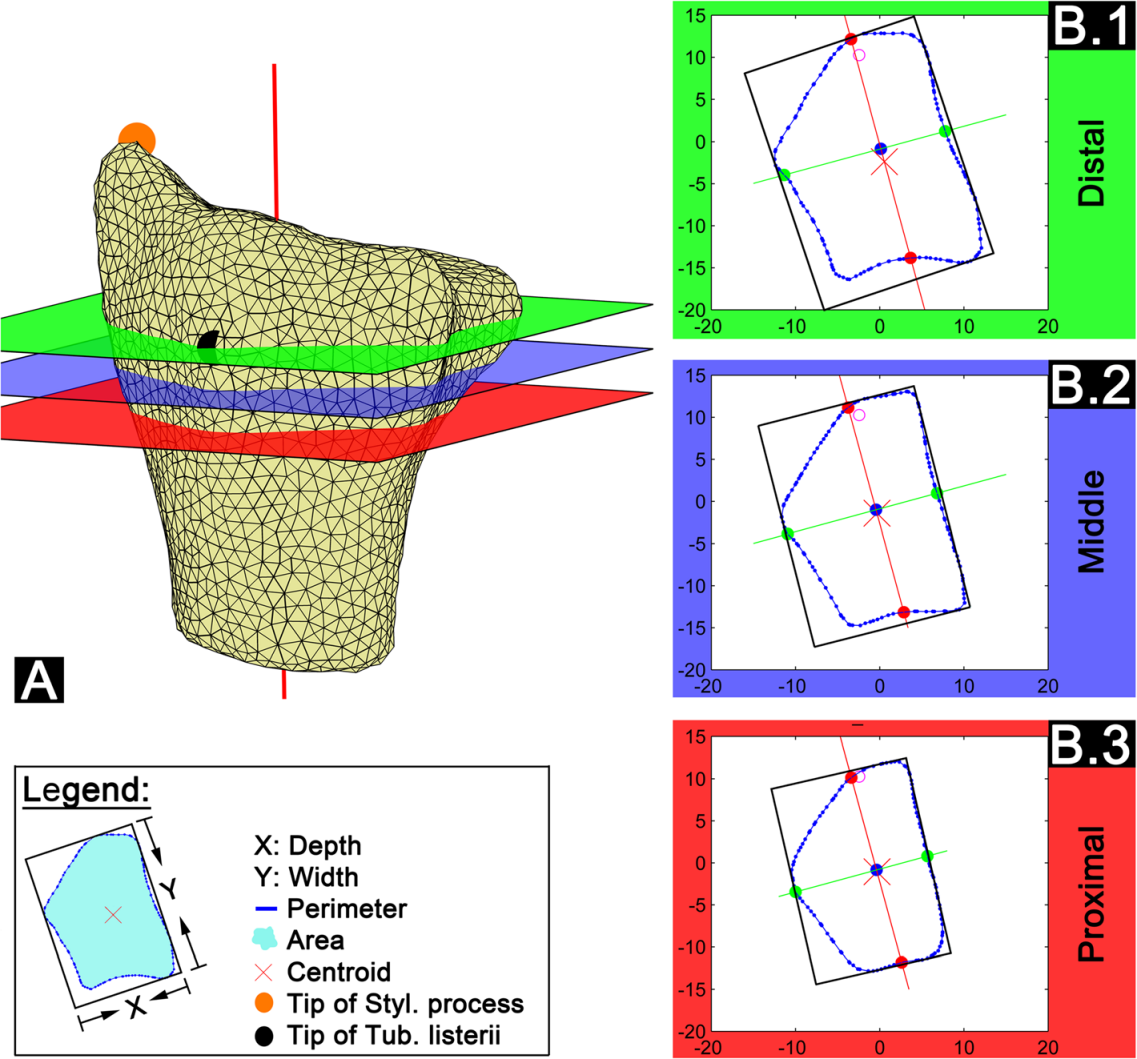
Illustration of the locations of the sectional planes and the assessed parameters. A) 3D reconstruction of the radius highlighting the cross-sectional cut planes; Styl. process: Styloid process; Tub. listerii: Tuberculum listerii; B) Exemplary presentation of the generated cross-sectional cut planes; B.1) Distal sectional plane at the most dorsal point of the tuberculum listerii; B.2) Middle sectional plane right in-between the Distal and Proximal plane; B.3) Proximal sectional plane 50% of the distance between the styloid process and the tuberculum listerii proximal to the Distal sectional plane

Three cross-sectional cut planes were defined for further morphometric analysis based on size-independent landmarks. These were the styloid process and the most dorsal point of the tuberculum listerii (Fig. 1 identification of landmarks and cut planes). Standardized three-dimensional sample orientation allowed the definition of cross-sectional cut planes perpendicular to the main axis. The following cross-sectional cut planes (Fig. 2) were chosen: *Distal*: The most dorsal point of the tuberculum listerii (Fig. 2B.1); *Proximal*: 50% of the distance between the tip of the styloid process and the most dorsal point of the tuberculum listerii (Fig. 2B.3); *Middle*: Half way between the distal and proximal sectional plane (Fig. 2B.2). Cross-sectional cut planes were calculated for the mean shape models as well as for each individual radius. An animated illustration of the cross-sectional cut planes is presented in Additional file 2. For each sectional plane the maximum width and depth, perimeter and area were calculated (Fig. 2 Legend).

### Classification accuracy for side and gender

The overall shape model was utilized to evaluate the predictive value for gender and side identification within our population. To do so leave-one-out cross validation (LOOCV) by repeatedly (100 iterations) removing one test shape and training a classifier (random forest with 50 decision trees) on the remaining shape model coefficients was conducted. The resulting classification accuracy was calculated. All computations were performed in Matlab R2015a.

### Statistics

Statistical differences in morphometric parameters (maximum width and depth, perimeter and area) between side and gender were calculated using SPSS 22.0 (IBM, Chicago, IL, USA). The Kolmogorov-Smirnov test was used to verify that data was normally distributed. General morphology statistics comprised of descriptive analysis, an independent sample *t*-Test and an ANOVA (post hoc test: Bonferroni). Due to multiple testing, a Bonferroni correction was conducted (*p* < 0.013). The ratio between correctly and incorrectly classified radii was used to calculate the classification accuracy of the overall shape model.

## Results

### Patient sample

One thousand two hundred nine radii were screened, 96 radii met the inclusion criteria. 1113 radii were excluded for the following reasons: fracture (*n* = 585), region of interest too small (*n* = 388), duplicates (*n* = 77), morphometric changes (*n* = 53), and age (*n* = 10). Ten more radii were excluded as they were used for pretests. The remaining 86 radii (45 left radii, 44% female, no pairs, mean age 40 ± 18 years (18–88 years)) were used to compute the models. The CT-indications for those radii were suspected distal radius- (25%) or suspected carpal fractures (75%).

### 3D shape model analysis

**Fig. 3:**
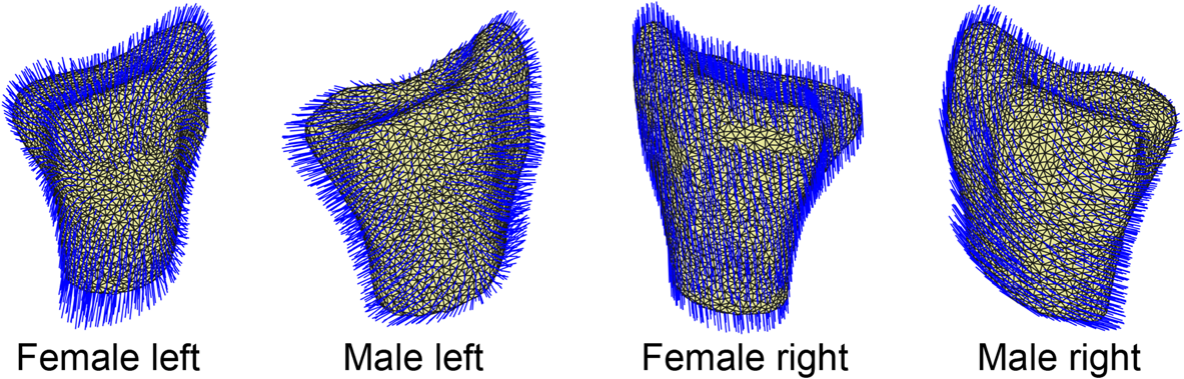
First mode of the four statistical shape models

All shape models were computed successfully. Figure 3 illustrates the gender and side specific models including their first mode. The first mode explained 37% of the overall variance and 41% / 34% / 25% / 50% of the variance of female / male / left / right radii respectively. Animated illustrations of the first five modes of all models are presented in Additional file 3A-D. Left radii had a higher shape variance (number of modes: 20 female / 23 male) compared to right radii (number of modes: 6 female / 6 male) (Additional file 4). Predominant shape variation directions (1. mode) for female radii were disto-proximal, while in the axial plane for the male left radii. The shape variation in male radii was curved, from lateral to medio-distal.

### Standardized cross-sectional cut planes and morphometric parameters

**Fig. 4:**
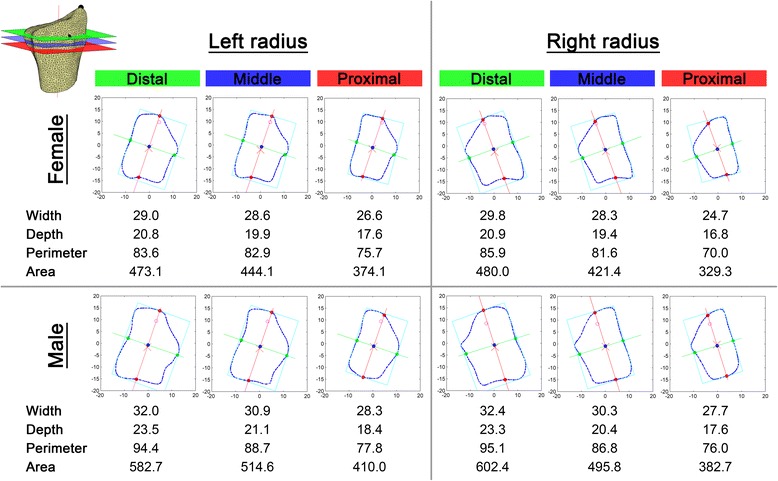
Cross-sectional cut planes of mean shape models and morphometric values. Length measures given in mm. Area measures given in mm^2^

Uniform cut planes were generated for the first mode of each mean shape model and every radius separately. Morphometric parameters, i.e. maximum width and depth, perimeter, and area (Fig. 2 Legend) were calculated. Figure 4 illustrates side and gender specific cross-sectional cut planes and their morphometric values for the mean shape model. The cross-sectional cut planes for ±1SD of the mean shape models are presented in Additional file 5, the subsequent morphometric values in Additional file 6. Overall, male radii were larger than female radii with no significant side difference. All morphometric parameters decreased from distal to proximal.

Furthermore, the three standardized cut planes were calculated for all 86 radii separately. The cross-sectional cut planes were on average 3.5 ± 0.6 mm apart from each other. Table 1 summarizes their descriptive morphometric parameters. All assessed plane parameters were significantly greater in male than in female patients (*p* ≤ 0.001). No significant side differences could be found. Comparing the morphometric parameters between the cross-sectional cut planes (ANOVA) gender separately revealed overall significant differences. The post-hoc analysis (Bonferroni) showed significant sectional plane differences for all parameters except female width, perimeter and area between the distal and middle sectional plane.

**Table 1:** Summary (mean +/- SD) of morphometric parameters of three different cross-sectional cut planes calculated directly from the CT slices (*n* = 86)

			Female	Female left	Female right	Male	Male left	Male right
Distal	Width	Mean ± SD	29.0 ± 2.1	28.7 ± 1.9	29.4 ± 2.4	33.0 ± 2.1	32.5 ± 2.1	33.5 ± 2.1
		Range	24.0–35.0	24.0–32.7	26.1–35.0	28.5–37.6	28.5–37.6	29.0–37.5
	Depth	Mean ± SD	21.0 ± 1.7	20.8 ± 1.7	21.2 ± 1.6	23.8 ± 1.4	23.5 ± 1.3	24.0 ± 1.5
		Range	17.3–24.7	17.3–24.7	18.9–24.6	21.0–26.3	21.8–26.3	21.0–26.2
	Perimeter	Mean ± SD	86.1 ± 6.1	84.9 ± 4.6	87.5 ± 7.4	96.5 ± 6.0	94.9 ± 5.8	98.2 ± 5.9
		Range	76.9–102.7	77.4–94.5	76.8–102.7	85.7–110.0	86.8–106.1	85.7–110.0
	Area	Mean ± SD	472.6 ± 70.2	464.0 ± 67.1	483.2 ± 74.5	593.1 ± 62.1	573.3 ± 57.4	612.9 ± 61.4
		Range	302.3–663.4	302.3–591.9	384.1–663.4	477.9–717.0	477.9–685.0	478.3–717.0
Middle	Width	Mean ± SD	28.2 ± 2.3	27.9 ± 1.9	28.6 ± 2.8	31.2 ± 2.2	30.9 ± 1.8	31.4 ± 2.6
		Range	24.3–34.2	24.6–31.9	24.3–34.2	25.9–37.5	27.4–34.8	25.9–37.5
	Depth	Mean ± SD	19.5 ± 1.8	19.4 ± 1.6	19.6 ± 2.0	21.6 ± 1.7	21.5 ± 1.4	21.7 ± 2.0
		Range	16.3–23.2	16.3–22.3	16.3–23.2	18.5–26.6	19.4–24.8	18.5–26.6
	Perimeter	Mean ± SD	81.5 ± 7.0	80.4 ± 5.4	82.9 ± 8.5	90.4 ± 6.7	89.3 ± 5.6	91.5 ± 7.6
		Range	68.5–99.5	72.7–91.2	68.5–99.5	75.1–107.5	79.5–100.8	75.1–107.5
	Area	Mean ± SD	427.5 ± 69.3	421.0 ± 60.8	435.6 ± 79.7	516.9 ± 67.7	507.2 ± 57.4	526.6 ± 76.6
		Range	315.5–586.9	337.6–556.9	315.5–586.9	372.3–706.9	427.3–669.1	372.3–706.9
Proximal	Width	Mean ± SD	26.3 ± 2.4	26.0 ± 2.1	26.5 ± 2.7	28.6 ± 2.4	28.5 ± 2.0	28.7 ± 2.7
		Range	21.4–31.8	21.4–30.2	22.0–31.8	23.4–35.8	24.9–33.3	23.4–35.8
	Depth	Mean ± SD	17.2 ± 1.7	17.2 ± 1.6	17.2 ± 1.9	18.6 ± 1.9	18.6 ± 1.6	18.7 ± 2.3
		Range	13.7–20.6	14.8–20.1	13.7–20.6	15.4–25.7	16.7–23.3	15.4–25.7
	Perimeter	Mean ± SD	73.4 ± 7.4	72.8 ± 6.3	74.1 ± 8.7	79.7 ± 7.7	79.1 ± 6.6	80.3 ± 8.8
		Range	59.9–92.1	63.1–85.3	59.9–92.1	64.1–106.0	70.1–97.7	64.1–106.0
	Area	Mean ± SD	353.2 ± 66.0	352.3 ± 63.9	354.3 ± 70.4	416.0 ± 71.4	410.7 ± 62.4	421.4 ± 80.4
		Range	242.7–486.8	254.2–486.8	242.7–479.2	280.7–640.8	328.1–612.0	280.7–640.8

Distal: Tuberculum dorsale; Proximal: ½ distance of proc. styl. rad. and the most dorsal point of the tub. dorsale; Middle: Plane in-between the disal and proximal plane

Finally, the morphometric parameters generated from the cut planes of the mean shape models were well within one standard deviation range of those of the individual radii values. The overall mean differences were distal: -0.8 ± 1.7 mm; middle: -1.0 ± 3.4 mm; proximal: -2.1 ± 4.4 mm.

### Classification accuracy of the overall shape model

**Fig. 5:**
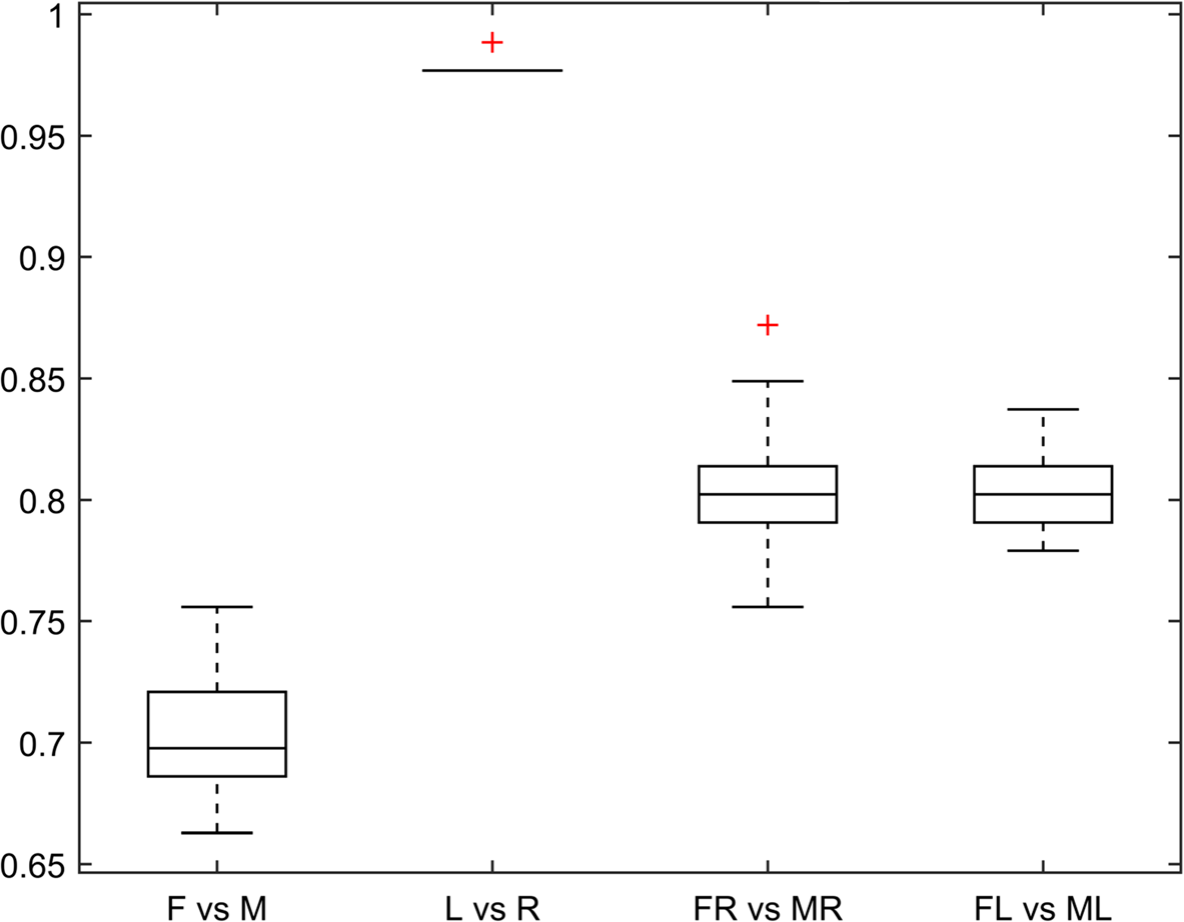
Predictive quality of the overall shape model. F: Female; M: Male; L: Left; R: Right

The overall shape model allowed accurate discrimination between left and right radii with a median classification probability of 98%. Testing for gender differences, 70% of the tested radii were classified correctly. Conducting the same analysis step-wise predictive, i.e. by firstly identifing the side, then the gender, yielded a median classification probability of 80% independent of the radii side. The predictive quality of the overall shape model is illustrated in Fig. 5.

## Discussion

A large dataset of clinical CT images was used to generate the first 3D mean shape model of the distal radius, define and analyze uniformly oriented cross-sectional cut planes, and finally provide good classification accuracy regarding side and gender.

Previous studies have predominantly been limited to the 2D anatomy of the distal radius. These assessed gross measurements (distal sagittal and axial width) [8–10, 25] or the cross-sectional anatomy of the distal radius [11, 26, 27]. Some studies indicated to assess the 3D anatomy of the distal radius. Hamilton et al. [27] analyzed various morphometric parameters at different locations of the distal radius. Oppermann et al. [11] analyzed longitudinal and transverse slices of 49 cadaver distal radii. Recent studies have attempted to analyze specific landmarks, such as the dorsal tubercle (Lister’s tubercle) [1, 28, 29], the volar surface [11, 18, 19], the promatory of the radius [5] or the radiocarpal surface [30]. However, these studies based their analysis on single slices.

This study is the first to approach the three-dimensional anatomy of the distal radius and examine its variability within a population. The morphometric parameters calculated from the mean shape model, correlated well with those from the individual cut planes. Moreover, these morphometric values mirror data from previous studies in similar populations [2, 8]. However, published morphometric distal radius values have shown a broad range, even within a population. This may either be due to a natural wide variability, or variations in measurement location between these studies.

This study highlights the significant influence of the measurement location on morphometric parameters. Although the generated cut-planes were only 3.5 ± 0.6 mm apart from each other on average, almost all morphometric parameters varied significantly. Therefore, two-dimensional analysis of cut-planes requires careful attention to the measurement location. Consistent measurement location may facilitate inter-study comparison. In future, however 3D anatomical models eliminate this confounder.

The retrospective dataset, generated from patients presenting with wrist/hand pain, is a limitation of this study. One may assume, that this patient cohort differs from a prospectively assessed, asymptomatic patient sample. However, patients who present at our level 1 trauma center are of an assumed random population. All CT-datasets were carefully reviewed by a fellow-trained orthopedic surgeon and radiologist to assure an intact bony architecture. Although trauma may have had an impact on the intra-osseos architecture, i.e. trabecular microfractures, we belive this most likely did not alter the cortical architecture of the distal radius. Therefore, as the cortical surface was used to generate the 3D-surface models, the initial trauma should not compromise the results.

One further point that warrants discussion is sexual dimorphism and the identification of handedness. A forensic study by Ruiz Mediavilla et al. [7] avoided the bias of measurement location by assessing the volume of 127 distal radii (twentieth century) to analyze the potential of volume measurements in order to determine the gender of fragmentary remains. For the distal radius, they reported significant greater values for male and right radii compared to female and left radii respectively. The current study, as well as previous studies, also found significant gender differences, but no such differences for side [11]. Moreover, Ruiz Mediavilla et al. [7] reported a gender classification function accuracy of their volumetric measurements of 95.5% for right and 88.5% for left radii. These values are higher than reported herein. This may either be due to our limited sample size or a greater gender difference in the volume rather than the shape.

Interestingly, a greater shape variance (number of modes) was determined in left compared to right radii. As no further quantitative analyses could be conducted on the total 3D shape models, we computed their cross-sectional cut planes (mean, ±1SD). The greater shape variance of left radii may be explained by the functional adaptation of bone to stress [31]. Previous studies showed a difference in metacarpal bone size depending on hand dominance [32]. With most of the population being right-handed, this could explain the herein observed shape variance between left and right radii.

3D surface models bear various advantages. Firstly, they are well established for the application in a medical context, such as assessing bone morphology [33–35], temporal-lobe morphology [36], or anthropometric shape evaluations of the human scalp [37]. Secondly, the methodology can quickly and efficiently process large data and generate 3D shape models based on clinical CT datasets. Therefore it may easily be adapted to various anatomical locations. Finally, although the overall shape variance could not be further analyzed quantitatively, this may be possible for specific anatomical landmarks. Future studies should, for example, assess the surface area and the 3D curve of the distal volar surface. This is of particular interest to the design of pre-shaped plates used in osteosynthesis in distal radius factures [3, 38].

## Conclusions

A novel 3D shape model of the distal radius was constructed allowing descriptive analysis of shape variance. Based on the shape model, uniform cut-planes were defined and analyzed. Assessment of the the model’s side and gender classification accuracy was 80%. Future studies may apply these models to other anatomical locations and assess specific anatomical landmarks.

## Additional files

Additional file 1: Detailed description of the process of shape model generation. (DOCX 92 kb)
Additional file 2: Animated illustration of the cross-sectional cut planes. Green plane: Proximal plane (50% of the distance between the tip of the styloid process and the most dorsal point of the tuberculum listerii); Blue plane: Middle plane (Half way between the distal and proximal sectional plane); Red plane: Distal plane (The most dorsal point of the tuberculum listerii). (PDF 140 kb)
Additional file 3: Animated illustrations of the first five modes of all radius models. A: Female left radii model; B: Female right radii model; C: Male left radii model; D: Male right radii model. (ZIP 9547 kb)
Additional file 4: Illustration of the shape variance (number of modes) for each radius model. (TIF 57327 kb)
Additional file 5: Illustration of the cross-sectional cut planes for ±1SD of the mean shape models. SD: Standard deviation; Distal: Distal plane; Middle: Middle plane; Proximal: Proximal plane. (TIF 9568 kb)
Additional file 6: Summary of morphometric parameters (Mean, ±1SD) of the mean shape model for all three sectional planes. SD: Standard deviation; Distal: Distal plane; Middle: Middle plane; Proximal: Proximal plane. (DOCX 113 kb)

## Abbreviations

2D: Two-dimensional
3D: Three-dimensional
CPD: Coherent point drift
CT: Computed tomography
DICOM: Digital imaging and communications in medicine
e: eigenvectors
Fig: Figure
l: Mean shape
LOOCV: Leave one out cross validation
n: number
PCA: Principle component analysis
SD: Standard deviation
Supp: Supplement
λ: Eigenvalues
