# Supplementary material for: Analysis of the three-dimensional anatomical variance of the distal radius using 3D shape models
Source: BMC Med Imaging. 2017 Mar 9;17:23. doi: 10.1186/s12880-017-0193-9 (PMC5343417; doi:10.1186/s12880-017-0193-9)
Supplement: Additional file 1: — Detailed description of the process of shape model generation. (DOCX 92 kb) [file 12880_2017_193_MOESM1_ESM.docx]

First, based on the 3D surface models, *n* 3-dimensional landmarks *l_i_=({x_1_,y_1_,z_1_}, …,{x_n_,y_n_,z_n_})* were defined for all *i ∈ 1, …, N* shapes based on the objects surface points. To ensure correspondence between those *n* landmarks Coherent Point Drift (CPD) is utilized {Myronenko, 2010, p17973}. The landmarks were then aligned using Procrustes Analysis by minimizing
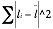
*,* with *l* representing the mean shape {Cootes, 2001, p17070}. The shape model is built by applying PCA on *L=(l_1_,…, l_n_)*, yielding Eigenvectors *e_1_, …, e_j_* and eigenvalues λ_1_, …, λ_j_ with *j=min(nd, N)*. Eigenmodes with the largest eigenvalues represent the highest shape variance. Modes with small variance are assumed to only model noise and were neglected. The aim was to capture 95% of the shape variance. Each shape within the generated subspace can be formulated by
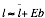
, where *E=(e_1_, …, e_j_); j’<j* and
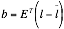
 is the model parameter vector. Additionally we accounted for similarity transformations (translation, scaling, rotation) by adding a parameter vector *t* resulting in the combined parameter vector *c=(b,t)* {Cootes, 2001, p17070}.
