# Supplementary figures and images for: Analysis of the three-dimensional anatomical variance of the distal radius using 3D shape models
Source: BMC Med Imaging. 2017 Mar 9;17:23. doi: 10.1186/s12880-017-0193-9 (PMC5343417; doi:10.1186/s12880-017-0193-9)

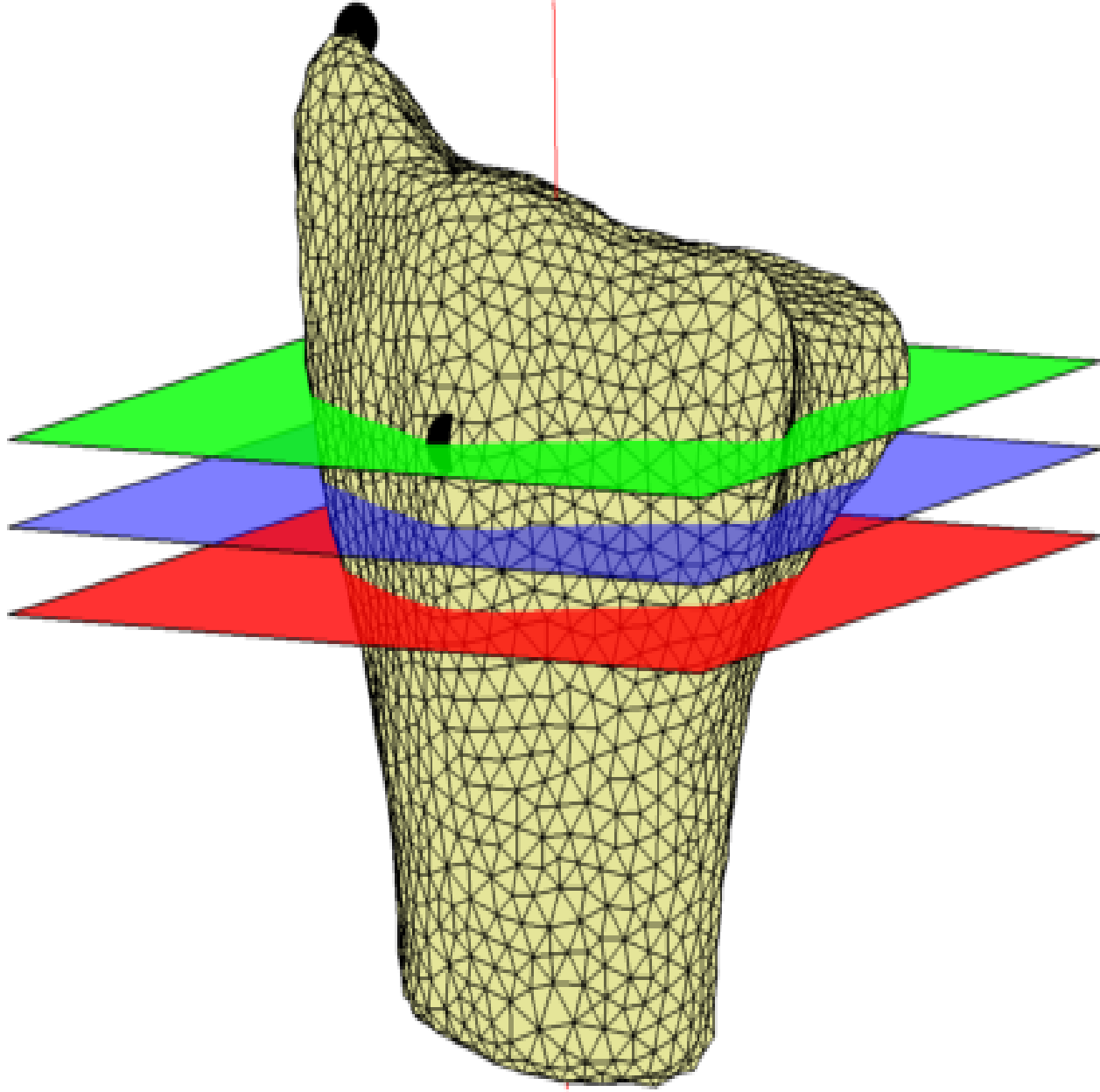

Supplement: Additional file 2: — Animated illustration of the cross-sectional cut planes. Green plane: Proximal plane (50% of the distance between the tip of the styloid process and the most dorsal point of the tuberculum listerii); Blue plane: Middle plane (Half way between the distal and proximal sectional plane); Red plane: Distal plane (The most dorsal point of the tuberculum listerii). (PDF 140 kb) [file 12880_2017_193_MOESM2_ESM.pdf]

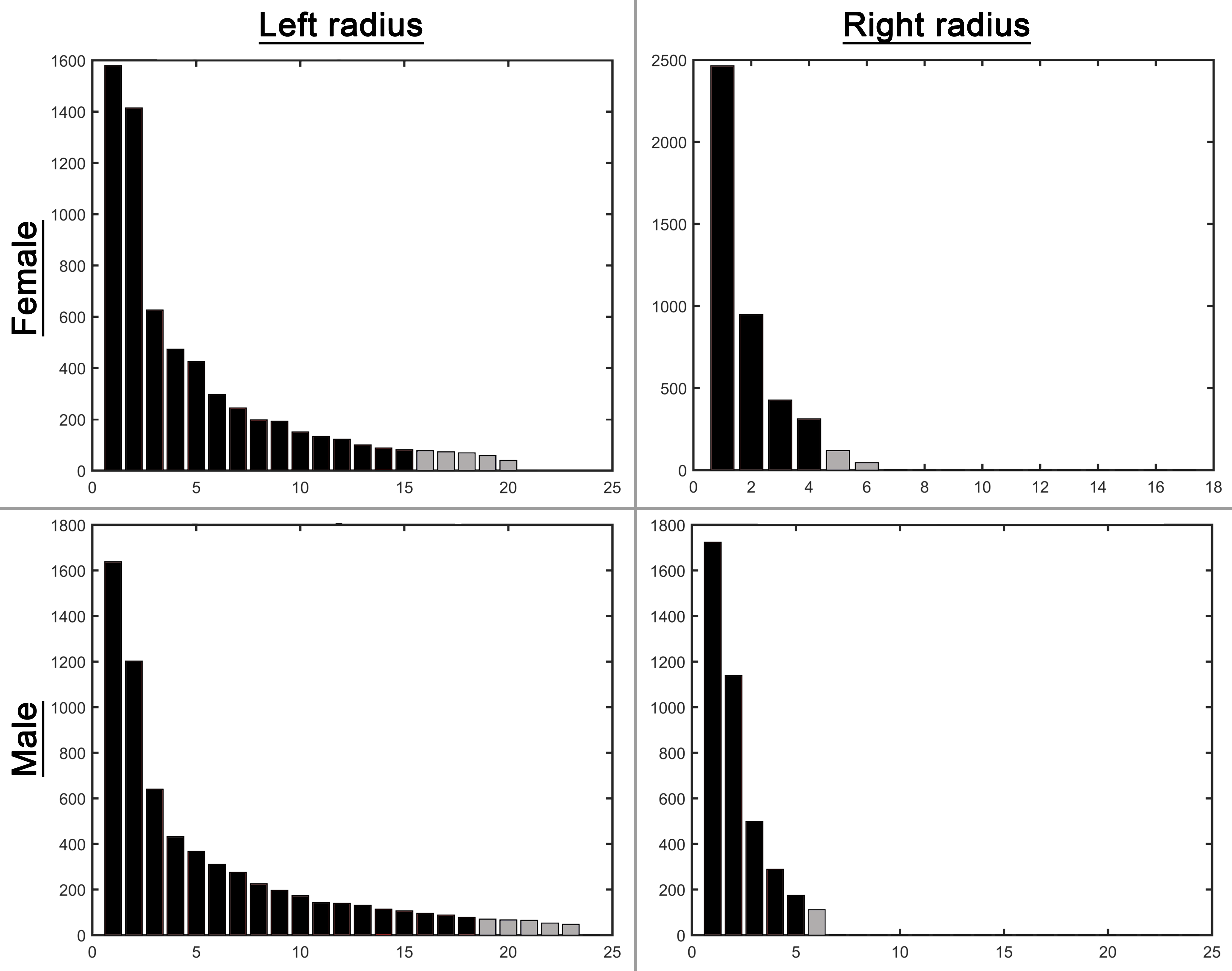

Supplement: Additional file 4: — Illustration of the shape variance (number of modes) for each radius model. (TIF 57327 kb) [file 12880_2017_193_MOESM4_ESM.tif]

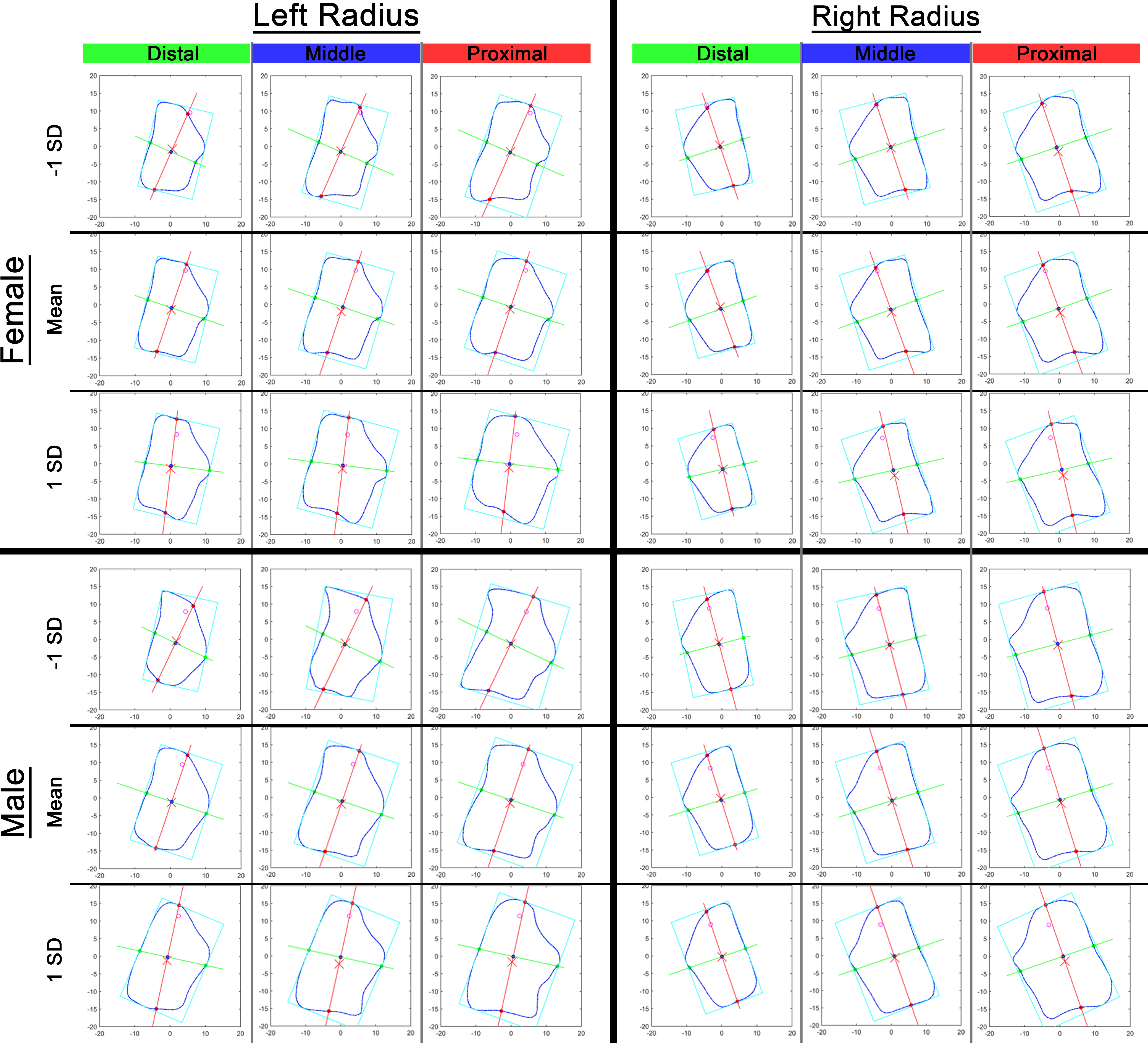

Supplement: Additional file 5: — Illustration of the cross-sectional cut planes for ±1SD of the mean shape models. SD: Standard deviation; Distal: Distal plane; Middle: Middle plane; Proximal: Proximal plane. (TIF 9568 kb) [file 12880_2017_193_MOESM5_ESM.tif]
