# Supplementary material for: Analysis of the three-dimensional anatomical variance of the distal radius using 3D shape models
Source: BMC Med Imaging. 2017 Mar 9;17:23. doi: 10.1186/s12880-017-0193-9 (PMC5343417; doi:10.1186/s12880-017-0193-9)
Supplement: Additional file 3: — Animated illustrations of the first five modes of all radius models. A: Female left radii model; B: Female right radii model; C: Male left radii model; D: Male right radii model. (ZIP 9547 kb) [file 12880_2017_193_MOESM3_ESM.zip › Supplement_3AR2.pdf]

Animated shape model modes for the female left radii model

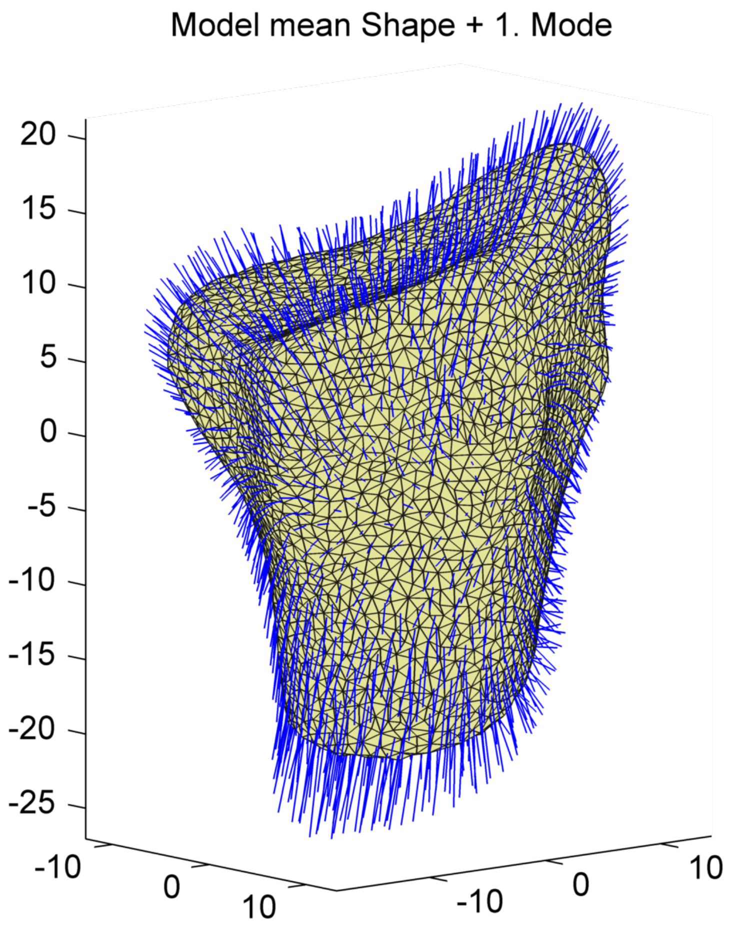

Figure 1: Mean shape of the female left radii model, including the first mode (drawn in blue).

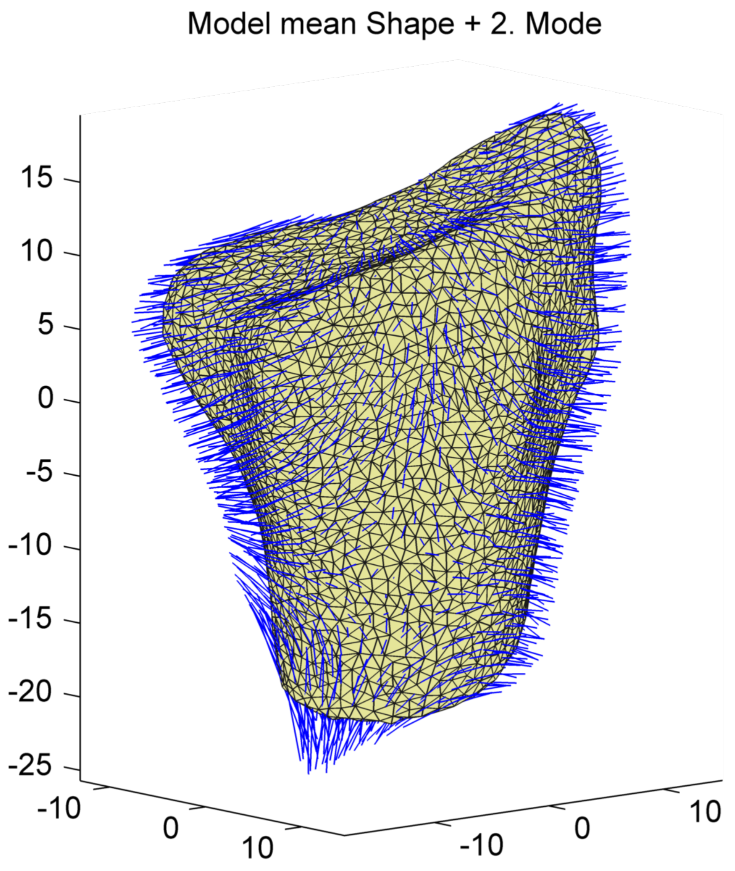

Figure 2: Mean shape of the female left radii model, including the second mode (drawn in blue).

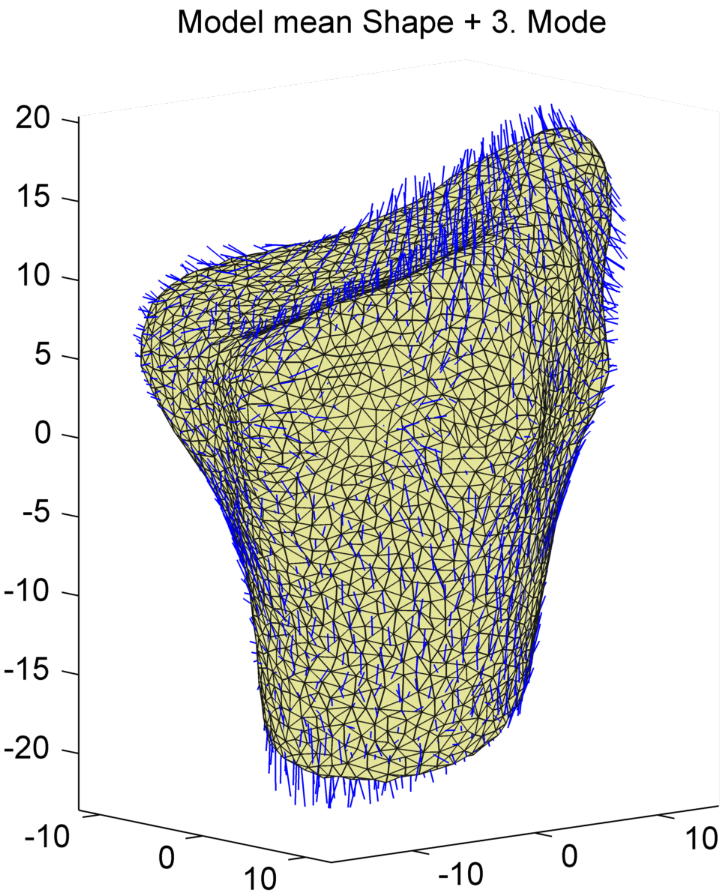

Figure 3: Mean shape of the female left radii model, including the third mode (drawn in blue).

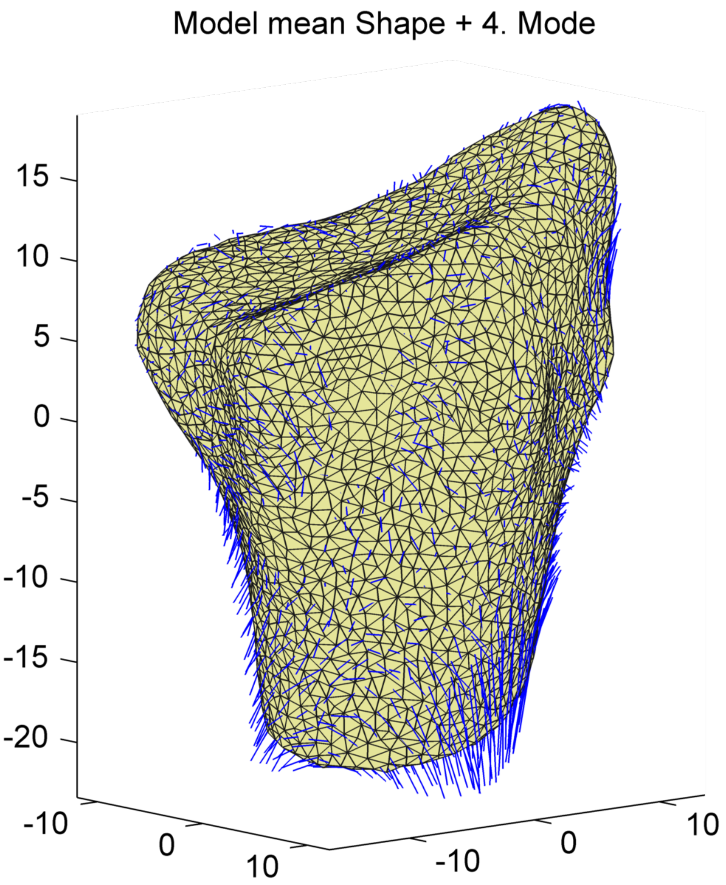

Figure 4: Mean shape of the female left radii model, including the fourth mode (drawn in blue).

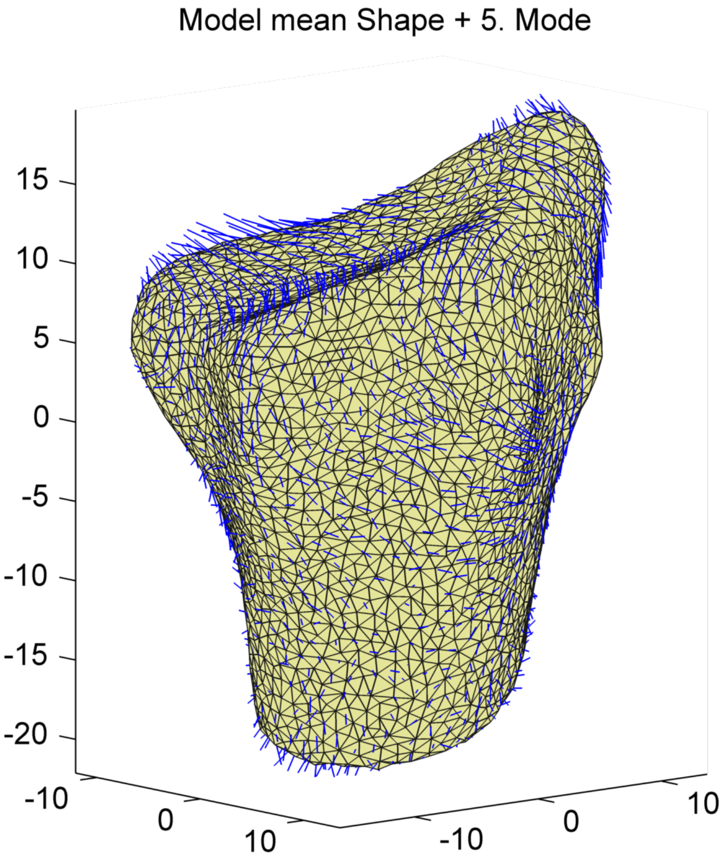

Figure 5: Mean shape of the female left radii model, including the fifth mode (drawn in blue).
