# Supplementary material for: Analysis of the three-dimensional anatomical variance of the distal radius using 3D shape models
Source: BMC Med Imaging. 2017 Mar 9;17:23. doi: 10.1186/s12880-017-0193-9 (PMC5343417; doi:10.1186/s12880-017-0193-9)
Supplement: Additional file 6: — Summary of morphometric parameters (Mean, ±1SD) of the mean shape model for all three sectional planes. SD: Standard deviation; Distal: Distal plane; Middle: Middle plane; Proximal: Proximal plane. (DOCX 113 kb) [file 12880_2017_193_MOESM6_ESM.docx]

| **Supplement 6:** Summary of morphometric parameters (Mean, ±1SD) of the mean shape model for all three sectional planes | | | | | | | |
| --- | --- | --- | --- | --- | --- | --- | --- |
|  |  |  | **Female left** | **Female right** |  | **Male left** | **Male right** |
| **Distal** | **Width** | +1SD | 29 | 29 |  | 34 | 33 |
|  |  | Mean | 29 | 30 |  | 32 | 32 |
|  |  | -1SD | 31 | 30 |  | 31 | 32 |
|  |  |  |  |  |  |  |  |
|  | **Depth** | +1SD | 23 | 21 |  | 23 | 23 |
|  |  | Mean | 21 | 21 |  | 24 | 23 |
|  |  | -1SD | 19 | 21 |  | 24 | 23 |
|  |  |  |  |  |  |  |  |
|  | **Perimeter** | +1SD | 86 | 86 |  | 96 | 96 |
|  |  | Mean | 84 | 86 |  | 94 | 95 |
|  |  | -1SD | 83 | 86 |  | 95 | 95 |
|  |  |  |  |  |  |  |  |
|  | **Area** | +1SD | 513 | 475 |  | 588 | 609 |
|  |  | Mean | 473 | 480 |  | 583 | 602 |
|  |  | -1SD | 431 | 481 |  | 539 | 606 |
|  |  |  |  |  |  |  |  |
| **Middle** | **Width** | +1SD | 29 | 28 |  | 33 | 30 |
|  |  | Mean | 29 | 28 |  | 31 | 30 |
|  |  | -1SD | 29 | 28 |  | 30 | 30 |
|  |  |  |  |  |  |  |  |
|  | **Depth** | +1SD | 22 | 19 |  | 21 | 20 |
|  |  | Mean | 20 | 19 |  | 21 | 20 |
|  |  | -1SD | 17 | 19 |  | 19 | 20 |
|  |  |  |  |  |  |  |  |
|  | **Perimeter** | +1SD | 86 | 80 |  | 92 | 87 |
|  |  | Mean | 83 | 82 |  | 89 | 87 |
|  |  | -1SD | 78 | 80 |  | 84 | 87 |
|  |  |  |  |  |  |  |  |
|  | **Area** | +1SD | 484 | 405 |  | 551 | 499 |
|  |  | Mean | 444 | 421 |  | 515 | 496 |
|  |  | -1SD | 380 | 416 |  | 418 | 500 |
|  |  |  |  |  |  |  |  |
| **Proximal** | **Width** | +1SD | 28 | 24 |  | 30 | 28 |
|  |  | Mean | 27 | 25 |  | 28 | 28 |
|  |  | -1SD | 25 | 25 |  | 26 | 28 |
|  |  |  |  |  |  |  |  |
|  | **Depth** | +1SD | 18 | 16 |  | 19 | 17 |
|  |  | Mean | 18 | 17 |  | 18 | 18 |
|  |  | -1SD | 16 | 17 |  | 16 | 18 |
|  |  |  |  |  |  |  |  |
|  | **Perimeter** | +1SD | 79 | 67 |  | 83 | 76 |
|  |  | Mean | 76 | 70 |  | 78 | 76 |
|  |  | -1SD | 69 | 70 |  | 69 | 76 |
|  |  |  |  |  |  |  |  |
|  | **Area** | +1SD | 399 | 306 |  | 465 | 382 |
|  |  | Mean | 374 | 329 |  | 410 | 383 |
|  |  | -1SD | 311 | 331 |  | 308 | 386 |
| Distal: Distal plane; Middle: Middle plane; Proximal: Proximal plane | | | | | | | |
